# Delays in health seeking, diagnosis and treatment for tuberculosis patients in Mongolia: an analysis of surveillance data, 2018–2021

**DOI:** 10.5365/wpsar.2024.15.1.1074

**Published:** 2024-02-21

**Authors:** Larissa Otero, Tsolmon Boldoo, Anuzaya Purevdagva, Uranchimeg Borgil, Temuulen Enebish, Oyunchimeg Erdenee, Tauhid Islam, Fukushi Morishita

**Affiliations:** a World Health Organization Regional Office for the Western Pacific, Manila, Philippines.; b Facultad de Medicina, Instituto de Medicina Tropical Alexander von Humboldt, Universidad Peruana Cayetano Heredia, Lima, Peru.; c National Tuberculosis Program, Ministry of Health, Ulaanbaatar, Mongolia.; d World Health Organization Representative Office for Mongolia, Ulaanbaatar, Mongolia.; e Global Tuberculosis Programme, World Health Organization, Geneva, Switzerland.

## Abstract

Early diagnosis and treatment of infectious tuberculosis (TB) is essential to the attainment of global targets specified in the End TB Strategy. Using case-based TB surveillance data, we analysed delays in health seeking, diagnosis and treatment among TB patients in Mongolia from 2018 to 2021. We calculated the median and interquartile range (IQR) for “diagnostic delay,” defined as the time from symptom onset to diagnosis, subdivided into “health-seeking delay” (time from symptom onset to first visit to a health facility) and “health facility diagnostic delay” (time from first health facility visit to diagnosis), and for “treatment delay,” defined as the time from diagnosis to start of treatment. We also calculated “total delay,” defined as the time from symptom onset to treatment start. Based on data for 13 968 registered TB patients, the median total delay was estimated to be 37 days (IQR, 19–76). This was mostly due to health-seeking delay (median, 23 days; IQR, 8–53); in contrast, health facility diagnostic delay and treatment delay were relatively short (median, 1 day; IQR, 0–7; median, 1 day; IQR, 0–7, respectively). In 2021, health-seeking delay did not differ significantly between men and women but was shorter in children than in adults and shorter in clinically diagnosed than in bacteriologically confirmed TB cases. Health-seeking delay was longest in the East region (median, 44.5 days; IQR, 20–87) and shortest in Ulaanbaatar (median, 9; IQR, 14–64). TB treatment delay was similar across sexes, age groups and types of TB diagnosis but slightly longer among retreated cases and people living in Ulaanbaatar. Efforts to reduce TB transmission in Mongolia should prioritize decreasing delays in health seeking.

Early diagnosis and prompt treatment of infectious tuberculosis (TB) cases reduce TB transmission and incidence and thus are crucial components of the End TB Strategy. (*1*) However, multiple barriers to early diagnosis and treatment exist and delays are common. Timely diagnosis requires individuals to recognize the symptoms of the disease and seek treatment. As TB symptoms such as cough and fever are common to other minor illnesses, individuals may delay seeking care until their symptoms become persistent or they develop additional symptoms (e.g. weight loss, night sweats). Diagnosing TB early is especially challenging in people with subclinical disease, who represent a potentially important subgroup to target, given recent evidence suggesting that half of bacteriologically confirmed TB cases are subclinical. (*2*)

People with TB symptoms often visit multiple informal and formal health services before a TB diagnosis is established. If a bacteriological test of a sputum specimen is positive, the case is confirmed, but if it is negative or the person cannot produce sputum, the diagnostic process can take longer because either a repeat sputum test or other evidence is required to establish a diagnosis. Limited access to health facilities and low levels of health knowledge and literacy are additional factors that can delay health seeking and diagnosis. (*3*)

Once a diagnosis has been made, prompt treatment is necessary to cure the disease and reduce transmission to others. Treatment initiation delays can arise because of poor health system organization, inefficient supply chains and lack of human resources. A global systematic review of studies found considerable heterogeneity in patient and health system delays for TB, (*4*) suggesting that time delays are setting-specific.

At 452 cases per 100 000 population, Mongolia has one of the highest TB incidence rates in the world. (*5*) Surveillance is conducted by the Mongolia National TB Programme and has established that in 2015–2019, the TB burden was heterogeneously distributed, with the country’s capital city, Ulaanbaatar, notifying more than half of all cases. (*6*) High notification rates among younger age groups suggest recent transmission, emphasizing the need to expand and accelerate case detection. (*6*) Previous studies have suggested that there is scope for improving TB diagnosis and treatment times across the country. A study in Ulaanbaatar conducted in 1996 reported that health-seeking delays averaged 29 days and health facility diagnostic delays averaged 35 days. (*7*) A later study, based on national data from 2016 and 2017, found that only 34% of patients were diagnosed and treated within 30 days of symptom onset. (*8*) Recent expansion of Xpert MTB/RIF testing nationwide in 2021 is expected to enhance case detection and reduce diagnostic delays.

In this report, we use national surveillance data for 2018–2021 to describe the time lapse between TB symptom onset and diagnosis, and between diagnosis and treatment initiation, in Mongolia. We also report diagnostic and treatment delays at the subnational level for 2021. Subnational analysis of diagnostic and treatment delays is needed to guide the planning of effective interventions tailored to local dynamics, as well as to monitor progress towards national and End TB Strategy targets and milestones.

## Methods

### Description of the surveillance system

In Mongolia, details of all registered TB cases are recorded on a patient treatment card by health-care workers at all health facilities that offer TB care. The card contains information such as the date of symptom onset (as reported by the patient), the date of diagnosis (defined as the date of bacteriological confirmation or the date on which a physician established a clinical–radiological TB diagnosis), and the date of TB treatment initiation. Most data from the patient treatment card are entered by health-care workers into TUBIS, an electronic case-based TB surveillance information system. The first version of TUBIS was implemented nationwide in 2012 and has since undergone significant development. Data quality is reviewed in quarterly meetings; oversight is provided by a data manager dedicated to TUBIS. Alongside TUBIS, the National TB Programme continues to operate its traditional aggregated data system, which provides quarterly reports on all notified cases of TB, from the basic management unit upwards. Over 95% of the aggregate data system notifications are also included in TUBIS. Patients treated for TB at the public hospital are not included in TUBIS.

### Analysis of surveillance data

**Fig. 1:**
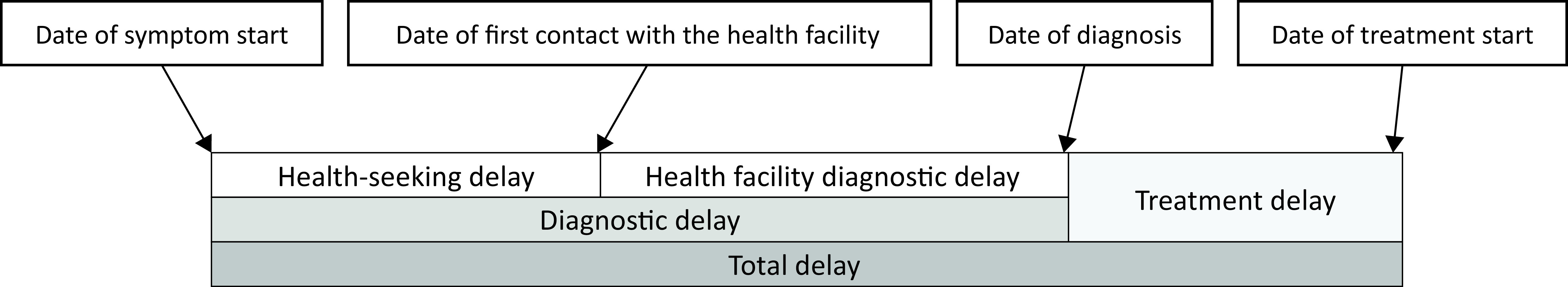
Definition of delays related to tuberculosis health seeking, diagnosis and treatment used in the study

For drug-susceptible TB cases captured by TUBIS from 2018 to 2021, we calculated the “diagnostic delay” (the time from symptom onset to diagnosis), the “treatment delay” (the time from diagnosis to treatment), and the “total delay” (the overall time from symptom onset to treatment). Diagnostic delay was further divided into “health-seeking delay,” defined as the time from symptom onset to first contact with a health facility, and “health facility diagnostic delay,” defined as the time from first health facility visit to diagnosis (**Fig. 1**).

[**insert** Figure 1]

In the absence of standard definitions of TB symptoms, specification of the time of symptom onset relied on inquiry by health staff. For asymptomatic cases identified by active case finding (i.e. through contact tracing or screening), the date of the screening test (sputum sample or chest X-ray) was used as the date of symptom onset. The date of diagnosis was the date of a positive bacteriological result or the date of a clinical or radiological diagnosis. Thus, for some cases the date of symptom onset and the date of diagnosis were the same.

Median and interquartile range (IQR) were calculated for each type of delay (in number of days) for the study period overall and for each of the four calendar years, 2018–2021. The statistical significance of yearly trends in each delay type was examined using the Mann–Kendall test. For 2021 cases, median delays were disaggregated by demographic (age, sex, place of residence) and clinical characteristics (type of diagnosis, type of case [new, relapse, other retreatment]). Mann–Whitney U and Kruskal–Wallis tests were used to compare differences in delays between groups of patients defined by these characteristics. Statistical significance was defined as *P* < 0.05.

Multivariable linear regression models were used to determine which patient characteristics were independently associated with longer diagnostic and treatment delays using a backward stepwise strategy, starting with a model that included all the variables that were significant in univariate analysis. Models were compared using the F test. Cases with implausible dates were excluded, i.e. cases in which the diagnosis date was before the time of symptom onset and those in which the treatment date was before the date of diagnosis. There were no records with missing dates. Data analysis was performed using STATA 17/SE software (Stata Corp, College Station, TX, USA).

## Results

After excluding 1779 (11.3%) case registrations with implausible dates (1634 for health-seeking delay and 145 for treatment delay), case-based data for 13 968 registered TB patients in Mongolia from 2018 to 2021 were analysed. There were 2718 registered TB cases in 2021, which represented 90.4% of all notified TB cases for that year.

Overall, the median (IQR) time lapse between symptom onset and initiation of treatment (total delay) was 37 (19–76) days. The median total diagnostic delay was 31 (14–66) days, whereas the median treatment delay was just 1 (0–7) day. Health-seeking delays (median, 23; IQR, 8–53) represented the greater contributor to diagnostic delays. There was no significant difference across the years in health-seeking delay (*P* = 0.89), nor was there any evidence of a trend in the total diagnostic delay (*P* = 0.12) (**Table 1**). However, there were significant upward trends over time in health facility diagnostic delay (*P* < 0.001), in treatment delay (*P* = 0.001) and in total delay (*P* = 0.009). The upward trend was most apparent in 2020 onwards and likely related to the impact of the COVID-19 pandemic.

**Table 1:** Tuberculosis diagnostic and treatment delays, Mongolia, 2018–2021

Year	Delay^a^ (days)					Proportion with total delay > 60 days (%)
	Health seeking	Health facility diagnostic	Total diagnostic	Treatment	Total	
**Overall**	23 (8–53)	1 (0–7)	31 (14–66)	1 (0–7)	37 (19–76)	32.6
2018	22 (8-–54)	1 (0–7)	30 (14–68)	1 (0–6)	36 (18–76)	32.2
2019	23 (9–52)	1 (0–8)	31 (14–64)	1 (0–7)	37 (19–74)	32.1
2020	23 (8–51)	2 (0–8)	31 (14–63)	2 (0–7)	38 (19–74)	31.9
2021	24 (9–56)	2 (0–8)	31 (15–69)	1 (0–7)	38 (21–82)	34.8
***P* test for** **annual trend** ^b^	0.89	< 0.001	0.12	0.001	0.009	-

^a^ Values are median (interquartile range) unless otherwise indicated.

^b^ *P*-values were calculated using the Mann–Kendall test.

In 2021, the total diagnostic delay was < 15 days in 35.7% of TB cases, 15–30 days in 23.4% of cases, 31–60 days in 17.1% of cases and > 60 days in 23.1% of cases. Total delay was < 15 days in 15.9% of TB cases; around a quarter of cases experienced total delays of 15–30 days (24.5%), another quarter had delays of 31–60 days (24.8%) and 34.8% of cases experienced delays of > 60 days (**Table 1**).

### Diagnostic delay

**Fig. 2:**
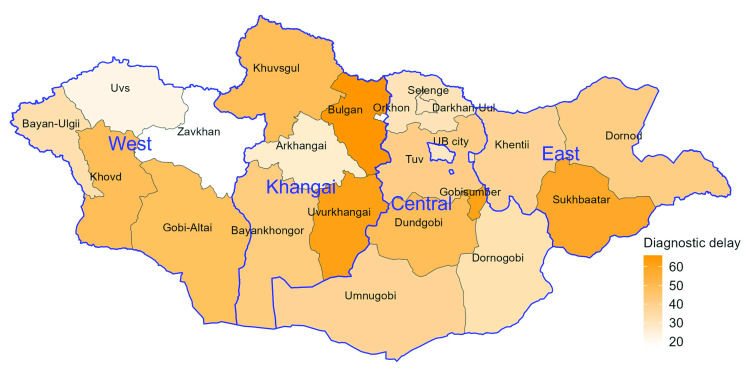
Median tuberculosis diagnostic delay (days) by province, Mongolia, 2021

Ulaanbaatar had the shortest median total diagnostic delay (29 days), followed by the West region (33 days) (**Table 2**). Both the East and Khangai regions had diagnostic delays higher than the national average (44.5, 41.5 and 31 days, respectively). There was heterogeneity in diagnostic delay within regions (**Fig. 2**). Fifteen provinces (five in Central, four in Khangai, three in East and three in West) had median diagnostic delays of 40 or more days from symptom onset.

[**insert** Figure 2]

**Table 2:** Tuberculosis diagnostic delay by patient characteristics and place of residence, Mongolia, 2021 (*n* = 2718)

Characteristic^a^	Health-seeking delay (days), median (IQR)	*P* ^b^	Health facility diagnostic delay (days), median (IQR)	*P* ^b^	Diagnostic delay (days), median (IQR)	*P* ^b^
**Total**	24 (9–56)		2 (0–8)		31 (15–69)	
**Sex**						
Female (*n* = 1 161)	22 (7–55)	**0.008**	2 (0–9)	0.251	31 (14–67)	0.11
Male (*n* = 1 557)	25 (10–56)		2 (0–7)		31 (17–70)	
**Age group**						
0–4 (*n* = 70)	4.5 (1–20)	**0.0001**	1 (0–7)	**0.048**	11.5 (3–26)	**0.0001**
5–14 (*n* = 223)	10 (3–23)		2 (0–8)		16 (6–31)	
15–24 (*n* = 512)	19 (7–41.5)		2 (0–7)		26 (13–55)	
25–34 (*n* = 655)	27 (10–62)		2 (0–7)		34 (17–79)	
35–44 (*n* = 442)	30 (10–63)		2 (0–9)		35 (20–78)	
45–54 (*n* = 411)	28 (11–68)		2 (0–7)		39 (19–86)	
55–64 (*n* = 270)	25 (11–66)		1 (0–8)		35 (20–90)	
≥ 65 (*n* = 133)	28 (11–53)		4 (0–14)		39 (22–77)	
**Type of diagnosis**						
Bacteriologically confirmed pulmonary TB (*n* = 1 453)	28 (11–61)	**0.0001**	2 (0–6)	0.085	35 (18–73)	**0.0001**
Clinically diagnosed pulmonary TB (*n* = 317)	18 (6–50)		2 (0–12)		27 (12–69)	
Extrapulmonary (*n* = 946)	19 (6–46)		2.5 (0–11)		28 (14–59)	
**Patient type**						
New (*n* = 30)	23 (8–53)	**0.0003**	2 (0–8)	0.815	30 (15–67)	**0.0001**
Relapse (*n* = 332)	25.5 (10–57)		2 (0–9.5)		33 (18–64)	
Other retreatment (*n* = 114)	37 (15–92)		2 (0–16.5)		60.5 (31.5–180.5)	
**Place of residence**						
**Ulaanbaatar (*n* = 1 829)**	22 (8–52)	**0.0004**	2 (0–8)	0.152	29 (14–64)	**0.0001**
**Khangai**	28 (10–65)	-	2 (0–10.5)	-	41.5 (18–91)	-
Arkhangai (*n* = 48)	25 (7.5–42)	-	0 (0–10)	-	27 (12–60.5)	-
Bayankhongor (*n* = 36)	31.5 (14–74.5)	-	2 (2–10.5)	-	42 (24.5–88)	-
Bulgan (*n* = 31)	57 (18–153)	-	4 (0–15)	-	66 (26–164)	-
Khuvsgul (*n* = 59)	13 (4–42)	-	8 (2–37)	-	49 (25.5–110)	-
Orkhon (*n* = 50)	31 (10–58)	-	1 (0–2)	-	18 (31.5–66)	-
Uvurkhangai (*n* = 32)	51.5 (25–115)	-	1 (0–4.5)	-	61.5 (25.5–115.5)	-
**Central**	29 (8–54)	-	2 (0–7.5)	-	35 (14.5–72.5)	-
Darkhan-Uul (*n* = 96)	21.5 (7–47.5)	-	1 (0–4.5)	-	30.5 (14–63)	-
Dornogovi (*n* = 44)	29.5 (7–57.5)	-	1.5 (1–7.5)	-	32.5 (11.5–66.5)	-
Dundgovi (*n* = 26)	37.5 (11–83)	-	5.5 (3–20)	-	49 (36–101)	-
Govisumber (*n* = 10)	29.5 (0–120)	-	1.5 (0–11)	-	61 (11–121)	-
Selenge (*n* = 85)	22 (5–46)	-	2 (0–9)	-	31 (12–63)	-
Tuv (*n* = 62)	35.5 (15–59)	-	0 (0–1)	-	40.5 (20–65)	-
Umnugobi (*n* = 21)	31 (9–43)	-	8 (3–21)	-	38 (22–74)	-
**East**	30 (13–75)	-	1 (0–8)	-	44.5 20–87	-
Dornod (*n* = 64)	24.5 (9–55)	-	3.5 (1–16.5)	-	41 (19–86)	-
Khentii (*n* = 74)	28.5 (17–72)	-	1 (0–15)	-	38 (19–80)	-
Sukhbaatar (*n* = 48)	50 (16–94.5)	-	0 (0–1.5)	-	58.5 (24.5–105.5)	-
**West**	27 (9–52)	-	4 (1–8)	-	33 (14–77)	-
Bayan-Ulgii (*n* = 33)	24 (7–71)	-	3 (0–13)	-	33 (16–85)	-
Gobi-Altai (*n* = 4)	47 (36–79)	-	0.5 (0–3)	-	47.5 (39–79)	-
Khovd (*n* = 28)	42 (17–76.5)	-	7 (3–15)	-	49 (25.5–110)	-
Uvs (*n* = 22)	19 (10–38)	-	1 (0–4)	-	22 (10–40)	-
Zavkhan (*n* = 12)	8 (5–17.5)	-	4 (1.5–8)	-	16 (11–26.5)	-

IQR: interquartile range.

**^a^** The Mann–Whitney U test was used to compare median delays between males and females; the Kruskal–Wallis test was used to compare median delays between groups of people categorized by age group, type of diagnosis, patient type and place of residence.

^b^ Significant *P*-values (< 0.05) are in bold.

In the multivariable analysis of health-seeking delays, both age and place of residence were significantly associated with time to first visit to a health-care facility; sex was not. Children (aged 0–4 and 5–14 years) had shorter delays than adults aged 25–34 years (*P* < 0.001 for both). Patients with bacteriologically confirmed pulmonary TB had a longer health-seeking delay compared with patients with clinically or radiologically diagnosed TB (*P* < 0.001); patients classified as “other retreatment” had longer delays than those classified as “new” cases (*P* < 0.001). All regions had longer median health-seeking delays than Ulaanbaatar, but this difference was only significant in the multivariable analysis for the East and Khangai regions (*P* = 0.024 and 0.013, respectively). Similar patterns were observed in the case of the multivariable analysis of total diagnostic delays.

### Treatment delay

**Fig. 3:**
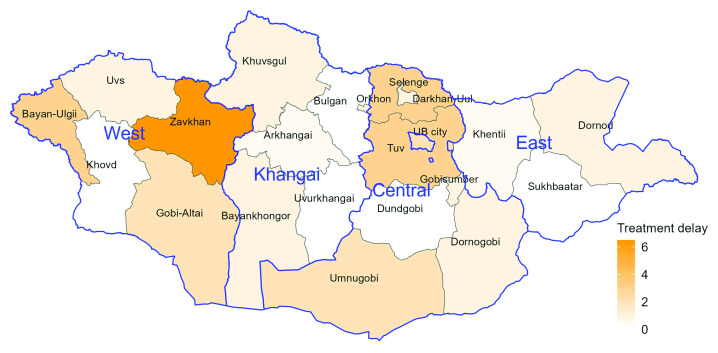
Median tuberculosis treatment delay (days) by province, Mongolia, 2021

Treatment delay did not differ significantly by sex or type of TB diagnosis (**Table 3**) and was less heterogeneous across provinces than was diagnostic delay (**Fig. 3**). Four provinces had median treatment delays of 3 or more days (Bayan-Ulgii, Selenge, Tuv and Zavkhan). The multivariable analysis of treatment delays suggested that children and young adults were more likely to start treatment sooner than older adults (*P* = 0.025). In addition, the time between diagnosis and treatment was significantly longer among “relapsed” cases (*P* = 0.016) and “other retreatment” cases (*P* < 0.001) than in “new” cases. Compared with Ulaanbaatar, treatment delays were significantly shorter in the East region (*P* = 0.012) and in the Khangai region (*P* = 0.049).

[**insert** Figure 3]

**Table 3:** Tuberculosis treatment and total delays by patient characteristics and place of residence, Mongolia, 2021 (*n* = 2718)

Characteristic^a^	Treatment delay (days), median (IQR)	*P* ^b^	Total delay (days), median (IQR)	*P* ^b^
**Total**	1 (0–7)	-	38 (21–82)	-
**Sex**				
Female (*n* = 1 161)	1 (0–7)	0.358	39 (19–79)	0.187
Male (*n* = 1 557)	1 (0–7)		38 (22–85)	-
**Age group**				
0–4 (*n* = 70)	1 (0–3)	**0.025**	15.5 (5–27)	**0.0001**
5–14 (*n* = 223)	1 (0–7)		24 (12–44)	
15–24 (*n* = 512)	1 (0–7)		32 (17–65)	
25–34 (*n* = 655)	2 (0–8)		45 (23–90)	
35–44 (*n* = 442)	2 (0–7)		45 (23–90)	
45–54 (*n* = 411)	2 (0–7)		50 (24–93)	
55–64 (*n* = 270)	2 (0–8)		46.5 (26–101)	
≥ 65 (*n* = 133)	1 (0–5)		48 (26–82)	
**Type of diagnosis**				
Bacteriologically confirmed pulmonary TB (*n* = 1 453)	1 (0–6)	0.757	42 (24–86)	**0.0001**
Clinically diagnosed pulmonary TB (*n* = 946)	1 (0–7)		34 (18–82)	
Extrapulmonary (*n* = 317)	1 (0–8)		33 (18–69)	
**Patient type**				
New (*n* = 30)	1 (0–7)	**0.0001**	36 (20–78)	**0.0001**
Relapse (*n* = 332)	2 (0–8)		45 (26–78.5)	
Other retreatment (*n* = 114)	4 (0–22)		123.5 (39.5–222.5)	
**Place of residence**				
**Ulaanbaatar (*n* = 1 829)**	2 (0–8)	**0.0001**	36 (21–78)	0.054
**Khangai**	1 (0–3)	-	46 (21–100)	-
Arkhangai (*n* = 48)	0.5 (0–8.5)	-	36 (20.5–75.5)	-
Bayankhongor (*n* = 36)	1 (0–7)	-	42.5 (24.5–93.5)	-
Bulgan (*n* = 31)	0 (0–6)	-	103 (28–164)	-
Khuvsgul (*n* = 59)	1 (0–3)	-	52 (17–100)	-
Orkhon (*n* = 50)	1 (0–2)	-	39.5 (21–67)	-
Uvurkhangai (*n* = 32)	0 (0–0)	-	67 (25.5–140)	-
**Central**	1 (0–5)	-	42 (20–82)	-
Darkhan-Uul (*n* = 96)	1 (0–2)	-	35 (17–72)	-
Dornogovi (*n* = 44)	1 (0–2)	-	41 (16.5–73)	-
Dundgovi (*n* = 26)	0 (0–4)	-	59 (40–136)	-
Govisumber (*n* = 10)	1 (1–7)	-	63 (19–122)	-
Selenge (*n* = 85)	3 (1–7)	-	34 (18–76)	-
Tuv (*n* = 62)	3 (0–13)	-	50.5 (31–101)	-
Umnugobi (*n* = 21)	2 (1–4)	-	46 (24–75)	-
**East**	1 (0–3)	-	48.5 (23–93)	-
Dornod (*n* = 64)	1 (0–3)	-	44 (19–90)	-
Khentii (*n* = 74)	0.5 (0–5)	-	41.5 (27–88)	-
Sukhbaatar (*n* = 48)	0 (0–1.5)	-	65 (28–111)	-
**West**	1 (0–5)	-	43 (19–90)	-
Bayan-Ulgii (*n* = 33)	3 (1–23)	-	54 (21–104)	-
Gobi-Altai (*n* = 4)	2 (0.5–90)	-	79.5 (49–159.5)	-
Khovd (*n* = 28)	0 (0–0)	-	49 (25.5–110)	-
Uvs (*n* = 22)	1 (0–1)	-	29 (12–44)	-
Zavkhan (*n* = 12)	6.5 (2.5–12)	-	23.5 (19.5–54)	-

IQR: interquartile range.

^a^ The Mann–Whitney U test was used to compare median delays between males and females; the Kruskal–Wallis test was used to compare median delays between groups of people categorized by age group, type of diagnosis, patient type and place of residence.

^b^ Significant *P*-values (< 0.05) are in bold.

## Discussion

Our analysis revealed that during the period of 2018–2021, the average time from symptom onset to treatment initiation for TB in Mongolia was 37 days. This was compounded mostly by diagnostic delay (median, 31 days), which in turn was mainly caused by health-seeking delay (median, 23 days). Health facility diagnostic delays contributed little to the overall diagnostic delay (median, 1 day). In 2021, time to diagnosis was shorter among children, but bacteriological diagnoses took longer than clinical and radiological diagnoses. Treatment delays were generally short (median, 1 day) but significantly longer among retreated TB cases. The East and Khangai regions had longer diagnostic delays but shorter treatment delays relative to Ulaanbaatar.

Our finding that delay in health seeking was the greatest contributor to the total delay is consistent with other studies, both global systematic reviews (*4*, *9*, *10*) and individual studies conducted in England, (*11*) Ethiopia (*12*) and India. (*3*) Our results are also similar to those obtained by a previous analysis of data from Mongolia for 2016–2017, which reported an average health-seeking delay of 28 days and an average health system delay (defined as health facility diagnostic delay plus treatment delay) of 7 days. (*8*) In addition, the proportion of TB patients with a health-seeking delay of more than 2 months (60 days) was similar to that reported in a 10-year analysis conducted in Japan (18% vs 21%, respectively). (*13*)

We attribute the shorter health-seeking delay that we observed among children to the fact that paediatric TB is frequently diagnosed through investigation of contacts and active case finding. We also found that among pulmonary cases, those that were clinically or radiologically diagnosed had shorter health-seeking delays than those that were bacteriologically diagnosed, suggesting that the former tend to present at an earlier stage of the disease; conversely, people whose TB disease takes longer to develop and who eventually become a bacteriologically confirmed case are more likely to delay seeking health care. This result is similar to that reported for Mongolia in 2016–2017, (*8*) and suggests that introduction of the sensitive rapid Xpert MTB/RIF test has yet to make a significant impact on both the number of bacteriologically confirmed cases and the length of diagnostic delays.

Our findings of similar delays in men and women, even after controlling for other variables, are consistent with a global systematic review that also found no differences by sex. (*4*) Other studies have identified low literacy (*12*, *14*, *15*) and first seeking care at informal providers (*4*, *15*) as factors that increase health-seeking delays, but we were not able to investigate these factors in our study.

We found that both health-seeking delays and treatment delays differed by geographical location. Generally speaking, health-seeking delays were shorter and treatment delays were longer in Ulaanbaatar compared with the rest of the country; these patterns were also evident in the earlier analysis of Mongolian surveillance data for 2016–2017. (*8*) It is likely that the shorter health-seeking delays in Ulaanbaatar, which were shorter in 2021 than in 1996 (22 and 29 days, respectively (*7*)), relate to easier access to health facilities and higher literacy rates in the capital. Such spatial heterogeneity is common in many countries regardless of income level, and multiple studies have shown that living in a rural area is associated with longer health-seeking delays. (*4*, *12*, *16*) Increasing access to health care through initiatives that deliver more patient-centred services (e.g. use of mobile teams in rural areas, adopting clinic opening hours that match patients’ preferences) has proven to reduce delays in health seeking. (*17*)

TB treatment delay in Mongolia was relatively short and similar to that reported by a study conducted in India. (*3*) Treatment delay was longer among relapsed and retreated cases, which may be explained by the need to first rule out drug resistance. The small but nevertheless significant longer treatment delay observed in Ulaanbaatar in 2021 compared with most regions and provinces may be a consequence of greater caseloads and more complex clinical and/or administrative steps and processes, resulting in longer wait times for treatment initiation. Outside the capital, where clinics are less busy, these might be completed more quickly. Only four provinces had median treatment delays longer than 2 days (Zavkhan, 6.5 days; Bayan-Ulgii, Selenge and Tuv, 3 days).

Use of routinely collected case-based data enabled us to analyse delays in TB diagnosis and treatment at both the national and subnational level. However, our study was limited by the lack of standardized definitions of TB symptoms and absence of data on several factors that are known to affect diagnostic delay, such as socioeconomic status and geographical access to health facilities. In addition, as data on treatment outcomes were rarely entered into TUBIS, we were unable to assess the impact of diagnostic and treatment delays on treatment outcomes. Finally, we did not include drug-resistant TB cases, which may have longer delays.

In sum, this study found that the lapse in time between initial symptom onset and start of treatment for TB (total delay) was dominated by the time taken by an individual to seek health care (health-seeking delay). Estimated median health-seeking delays were either comparable or lower than those documented in other settings and to those reported in Mongolia in 2016–2017 and in the 1990s. However, evidence of persistent longer health-seeking delays at regional and provincial levels highlights the need to increase access to TB diagnostic health facilities. Strategies such as community education and awareness programmes, same-day diagnosis and effective use of specimen transportation mechanisms and mobile heath teams could help reduce diagnostic delays in rural areas. Enhanced or active case finding of bacteriologically confirmed cases, including increasing the availability of Xpert MTB/RIF tests, could help reduce diagnostic delays and transmission. Finally, including data on socioeconomic and other factors that affect health-seeking behaviours in routine TB surveillance would improve our understanding of the causes of delays.
